# Targeting of BMI-1 expression by the novel small molecule PTC596 in mantle cell lymphoma

**DOI:** 10.18632/oncotarget.25558

**Published:** 2018-06-19

**Authors:** Aya Maeda, Yuki Nishida, Marla Weetall, Liangxian Cao, Arthur Branstrom, Jo Ishizawa, Takenobu Nii, Wendy D. Schober, Yoshiaki Abe, Kosei Matsue, Mariko Yoshimura, Shinya Kimura, Kensuke Kojima

**Affiliations:** ^1^ Division of Hematology, Respiratory Medicine and Oncology, Department of Internal Medicine, Saga University, Saga, Japan; ^2^ PTC Therapeutics, South Plainfield, NJ, USA; ^3^ Section of Molecular Hematology and Therapy, Department of Leukemia, The University of Texas MD Anderson Cancer Center, Houston, TX, USA; ^4^ Division of Hematology/Oncology, Department of Medicine, Kameda Medical Center, Kamogawa, Japan

**Keywords:** BMI-1, mantle cell lymphoma

## Abstract

Despite the development of the novel Bruton tyrosine kinase inhibitor ibrutinib, mantle cell lymphoma (MCL) remains an incurable B-cell non-Hodgkin lymphoma. BMI-1 is required for the self-renewal and maintenance of MCL-initiating stem cells. Upregulation of BMI-1 has been reported in MCL patients, especially in those with refractory/relapsed disease. We studied the effects of a novel small-molecule selective inhibitor of BMI1 expression, PTC596, in MCL cells. Eight MCL cell lines and patient-derived samples were exposed to PTC596. PTC596 induced mitochondrial apoptosis, as evidenced by loss of mitochondrial membrane potential, caspase-3 cleavage, BAX activation, and phosphatidylserine externalization. There was a positive correlation between baseline BMI-1 protein levels and PTC596-induced apoptosis. p53 status did not affect sensitivity to PTC596. PTC596 effectively decreased BMI-1-expressing and tumor-initiating side population MCL cells (IC_50_: 138 nM) compared with ibrutinib, which modestly decreased side population cells. Interestingly, PTC596, reported to target cancer stem cells, decreased MCL-1 expression levels and antagonized ibrutinib-induced increase in MCL-1 expression, leading to synergistic apoptosis induction in MCL cells. There are currently no drugs that specifically target cancer stem cell fractions, and a reduction in BMI-1 protein by PTC596 may offer a novel therapeutic strategy for MCL.

## INTRODUCTION

Mantle cell lymphoma (MCL) is an aggressive B-cell non-Hodgkin lymphoma typically associated with the t(11;14) translocation, resulting in overexpression of cyclin D1 (*CCND1*) [1]. Although the majority of patients with MCL remain incurable, the first-in-class Bruton tyrosine kinase (BTK) inhibitor ibrutinib has provided a new treatment option [2, 3]. Current studies aim to optimize the role of ibrutinib in MCL therapy and to address chemoresistance [4–6]. B-cell specific Moloney murine leukemia virus integration site 1 (BMI-1) is implicated in self-renewal and maintenance of tumor-initiating cancer stem cells in various malignancies [7, 8]. BMI-1 has been found to be upregulated in MCL cell lines and patient-derived MCL cells, most prominently in side population (SP) cells that have high *in vivo* tumorigenicity and self-renewal capability [9–11]. For example, SP cells, as defined by Hoechst dye exclusion in flow cytometry, have been identified in the MCL cell line REC-1, where BMI-1 is highly expressed compared to non-SP cells [9]. In a serial transplantation assay, the REC-1 SP cells have been found to generate tumors in primary, secondary and tertiary transplantation, whereas the non-SP cells lost tumorigenic potential after the primary transplantation. Therefore, the MCL SP cells have been thought to be enriched in cells with tumor-initiating stem-like characteristics. Importantly, BMI-1 levels in MCL cells have been found to be higher in refractory/relapsed patients than those at initial diagnosis [9]. Multiple pathogenic mechanisms appear to contribute to BMI-1 overexpression. The *BMI1* gene is amplified in approximately 10% of MCL cases, and the remainder show high mRNA and protein levels of BMI-1 without gene amplification [10].

PTC-209 and PTC-028/PTC596 are recently-developed novel small-molecule selective inhibitors of BMI1 expression that exhibit distinct modes of action [12–14]. PTC-209 has been reported to interfere with post-transcriptional regulation of BMI-1 and down-regulate BMI-1 production [12]. On the other hand, PTC-028 and its clinical analog PTC596 induce phosphorylation of BMI-1 at two N-terminal sites, leading to accelerated degradation of BMI-1 [13–16]. Although the preclinical utility of PTC-209 has been described in many cancers [12, 17–21], it has not entered clinical trials because of its limited potency and poor pharmacokinetic properties. The newer and potent compound PTC596 has completed a Phase 1 clinical trial in patients with advanced solid tumors (NCT02404480), showing a favorable safety profile [22]. The recommended Phase 2 dose was also determined (7 mg/kg orally twice a week). PTC596 has been reported to efficiently kill patient-derived CD34^+^CD38^low/−^ stem/progenitor cells in acute myeloid leukemia (AML) [14].

In this study, we investigated the anti-MCL effects of PTC-209 and PTC596, focusing particularly on PTC596, which is currently in clinical development.

## RESULTS

### PTC596 and PTC-209 exhibit p53-independent anti-MCL effects and high BMI-1 levels correlate with increased susceptibility to PTC596

**Figure 1:**
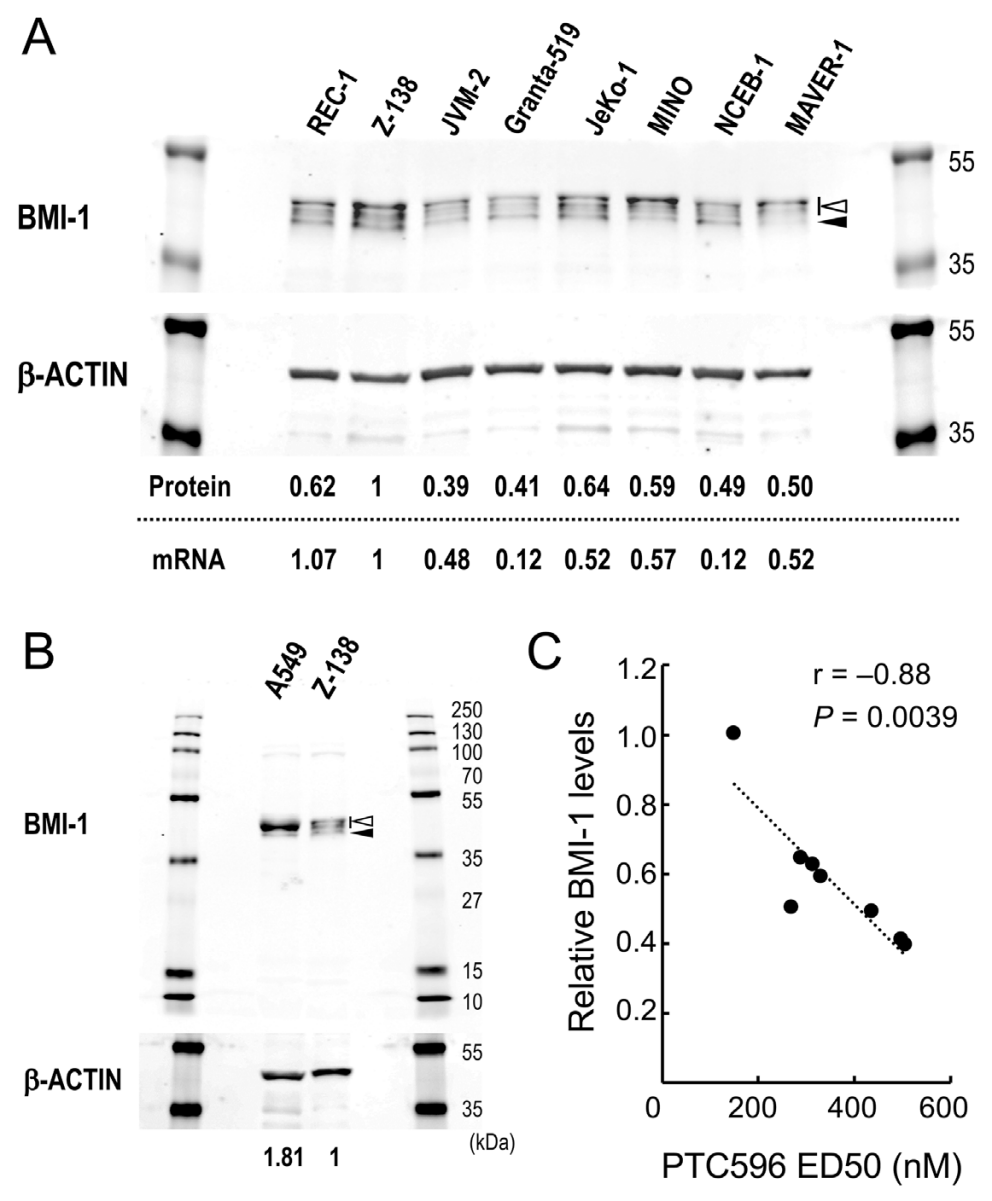
Basal levels of BMI-1 determine the sensitivity of mantle cell lymphoma (MCL) cells to the BMI-1 inhibitor PTC596 **(A)** Basal protein and mRNA expression levels of BMI-1 in MCL cell lines. The intensities of immunoblot signals were quantified and normalized to those of β-ACTIN. Levels in Z-138 cells were set at 1.0. Slow migrating phosphorylated BMI-1 bands are indicated by the open arrowhead, and non-phosphorylated BMI-1 is indicated by the closed arrowhead. mRNA expression levels of *BMI1* were quantitated by real-time PCR. **(B)** Basal protein expression levels of BMI-1 in lung cancer cell line A549 cells that express high levels of BMI-1 and Z-138 cells. **(C)** Correlation coefficient and probability values of ED_50_ values for PTC596 relative to BMI-1 protein levels.

We first examined the effect of PTC-209 and PTC596 on the proliferation and viability of cultured MCL cell lines. PTC-209 and PTC596 inhibited cell proliferation and induced apoptosis in a dose- and time-dependent manner. IC_50_ values at 72 hours ranged from 1.5 to 11.2 μM for PTC-209 and from 68 to 340 nM for PTC596 (Table 1). ED_50_ values at 72 hours ranged from 2.7 to > 50 μM for PTC-209 and from 150 to 507 nM for PTC596. PTC596 was > 10 times more potent than PTC-209. IC_50_ and ED_50_ values of PTC-209 positively correlated with those of PTC596 [r = 0.94 (*P* = 0.0004) for IC_50_ and r = 0.85 (*P* = 0.015) for ED_50_], respectively, supporting the idea that the anti-lymphoma activities of PTC-209 and PTC596 primarily depend on inhibition of BMI-1 expression. Importantly, high BMI-1 protein levels predicted high sensitivity to the clinical stage compound PTC596 (r = -0.88; *P* = 0.0039) (Figure 1). There was a positive correlation between BMI-1 protein levels and its mRNA levels in MCL cell lines (*r* = 0.71; *P* = 0.047) (Figure 1A), and high BMI-1 mRNA levels also predicted high sensitivity to PTC596 (r = -0.73; *P* = 0.042).

**Table 1:** Anti-proliferative and apoptotic effects of PTC-209 and PTC596 in mantle cell lymphoma (MCL) cell lines

	IC50		ED50	
	PTC-209 (μM)	PTC596 (nM)	PTC-209 (μM)	PTC596 (nM)
REC-1	2.7	136	6.7	314
NCEB-1	11.2	340	>50	438
MINO	2.5	122	9.9	331
MAVER-1	2.8	144	5.0	271
JVM-2	3.0	208	21.6	507
Granta-519	9.2	251	22.5	498
JeKo-1	0.9	68	2.7	291
Z-138	1.5	95	7.6	150

IC50, inhibitory concentration at which cell proliferation is inhibited by 50% as determined by trypan blue dye exclusion assay; ED50, effective concentration inducing 50% killing as measured by annexin V positivity.

BMI-1 resides upstream of ARF in ARF–MDM2–p53 signaling and therefore we postulated that the activity of PTC-209 and PTC596 depends on functional p53 to induce apoptosis in MCL cells. We took advantage of the clinical compound, PTC596, that is an inhibitor of BMI-1 expression, instead of PTC-209. ED_50_ values in p53 wild-type cells (Z-138, JVM-2 and Granta-519) were not significantly different to those in p53 mutant cells (MINO, JeKo-1, REC-1, MAVER-1 and NCEB-1) (384.8 ± 117.7 nM versus 328.8 ± 29.1 nM; *P* = 0.57). ED_50_ values were also determined in Z-138 and JVM-2 cells that stably express *TP53*-specific shRNA [23]. *TP53*-specific shRNA reduced p53 levels by approximately 90% (Supplementary Figure 1). The ED_50_ values were not significantly different between the control and p53 knockdown cells (Table 2). These data suggest that p53 signaling is not pivotal in PTC596-induced apoptosis.

**Table 2:** Effective doses of PTC596 for inducing 50% killing (ED50) in p53 wild-type MCL cells expressing either control shRNA or *TP53*-specific shRNA

Cell	shRNA	ED50 (nM)
Z-138	control shRNA	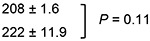
	*TP53*-specific shRNA	
JVM-2	control shRNA	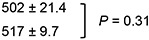
	*TP53*-specific shRNA	

ED50, effective concentration inducing 50% killing as measured by annexin V positivity.

### PTC596 induces mitochondrial apoptosis in MCL cells through down-regulation of MCL-1

**Figure 2:**
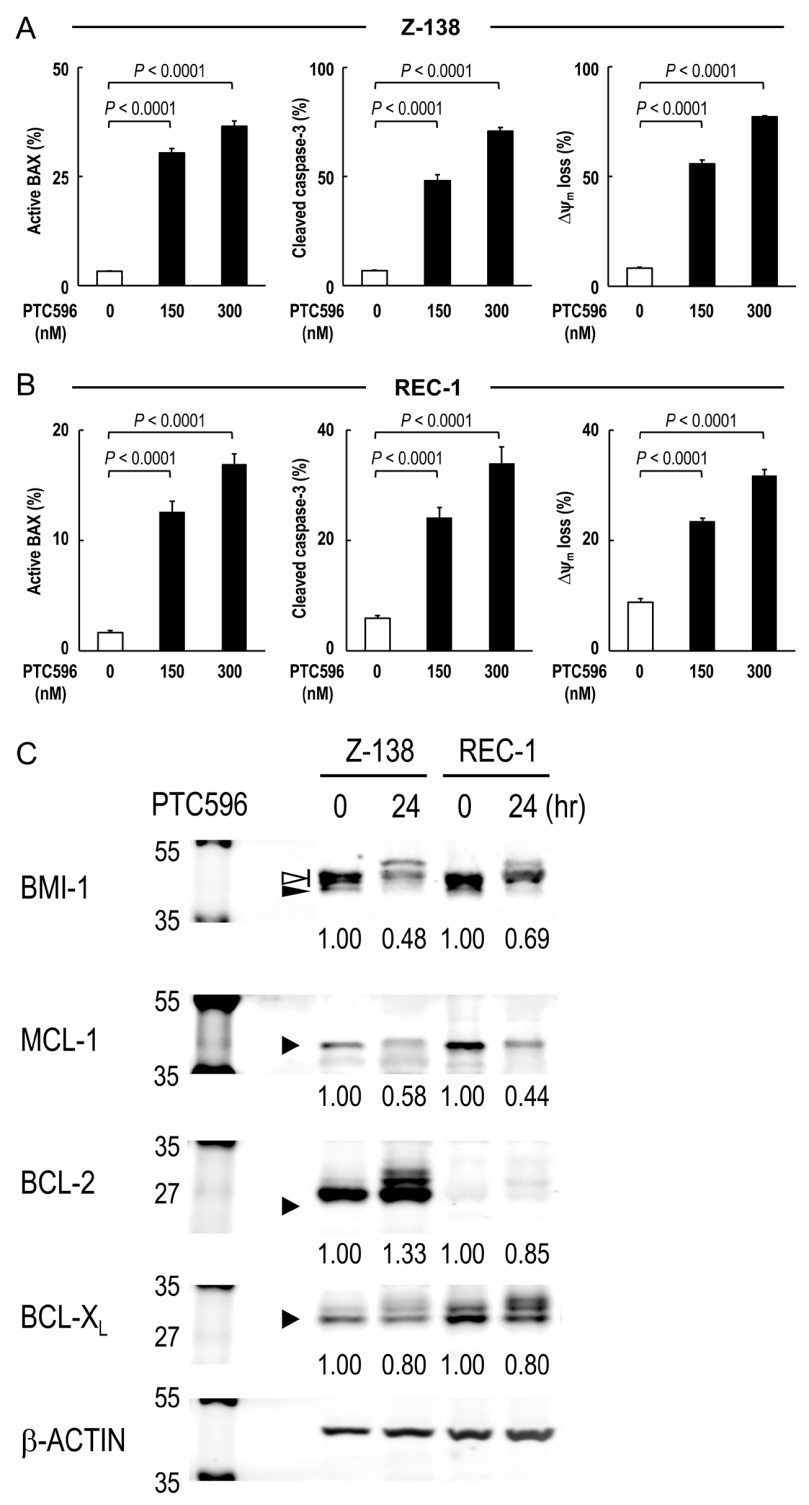
PTC596 induces mitochondrial apoptosis in mantle cell lymphoma cells **(A-B)** BAX conformational changes, caspase-3 cleavage and Δψm loss were determined by flow cytometry in Z-138 cells (A) or REC-1 cells (B) after 18-h (BAX activation) or 20-h (caspase-3 cleavage and Δψ_m_ loss) exposure to PTC596. The results are expressed as the mean ± SD. **(C)** Z-138 and REC-1 cells were treated for 24 hours with 300 nM PTC596, after which BCL-2, BCL-X_L_ and MCL-1 protein levels were determined. In BMI-1, slow migrating phosphorylated BMI-1 bands are indicated by the open arrowhead, and non-phosphorylated BMI-1 is indicated by the closed arrowhead. The intensities of immunoblot signals were quantified and normalized to those of β-ACTIN. Levels in untreated cells were set at 1.0. Results are representative of three independent experiments.

To investigate the involvement of the intrinsic pathway in PTC596-induced apoptosis, BAX conformational changes, caspase-3 cleavage and Δψ_m_ loss were determined in Z-138 and REC-1 cells after exposure to PTC596. PTC596 induced BAX activation, caspase-3 cleavage and ΔΨ_m_ loss (Figure 2A, 2B) in parallel with phosphatidylserine externalization data (Table 1), indicating that PTC596 kills MCL cells primarily through activation of the intrinsic apoptotic pathway. Since anti-apoptotic BCL-2 family proteins maintain mitochondrial membrane potential, changes in MCL-1, BCL-2, and BCL-X_L_ protein levels were determined in Z-138 and REC-1 cells. PTC596 decreased MCL-1 levels in both cell lines (Figure 2C). Changes in MCL-1, BCL-2, and BCL-X_L_ protein levels were further determined in six additional MCL cell lines. Again, PTC596 decreased MCL-1 levels in all six cell lines (Supplementary Figure 2). The PTC596-induced reduction of BMI-1 levels positively correlated with that of MCL-1 in eight MCL cell lines (*r* = 0.70; *P* = 0.045). On the other hand, changes were inconsistent in BCL-2 and modest in BCL-X_L_ among cell lines. To investigate the possibility that decrease in MCL-1 is attributable to its caspase cleavage secondary to apoptosis, time course changes in PARP and MCL-1 were determined in Z-138 cells under PTC596 exposure in the presence or absence of the caspase inhibitor Z-VAD. Cleavage of PARP by caspases is considered to be a hallmark of apoptosis. As shown in Supplementary Figure 3, the decrease in MCL-1 expression appeared to precede the PARP cleavage. In addition, Z-VAD partially inhibited PARP cleavage but it did not significantly affect reduction of MCL-1 expression. The data suggested that PTC596 downregulates MCL-1 and induces mitochondrial apoptosis.

### PTC596 decreases the number of MCL SP cells

**Figure 3:**
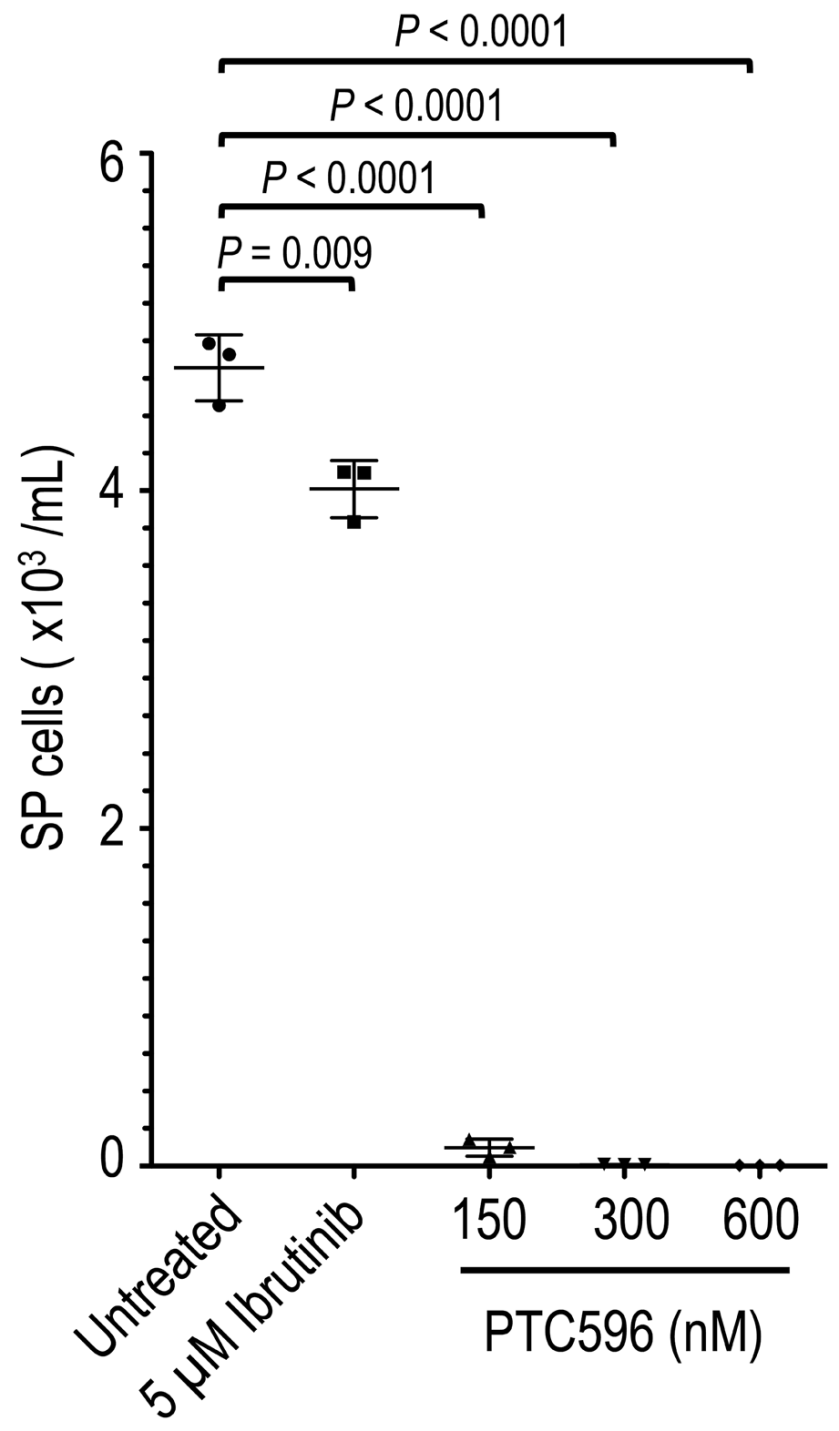
PTC596 deplete MCL SP cells REC-1 cells at an initial density of 4 × 10^5^ cell/mL were incubated with the indicated concentrations of ibrutinib or PTC596 for 72 hours, and the side population was analyzed based on the exclusion of Hoechst 33342. The results are expressed as the mean ± SD. Representative flow cytometric dot plots are also shown.

It has been reported that SP cells in a subset of MCL cell lines exhibit tumor-initiating characteristics with high tumorigenicity and self-renewal capability [9]. We screened for SP cells in eight MCL cell lines based on their ability to export Hoechst 33342. We reproducibly detected a SP fraction in REC-1 but not in the other seven cell lines. SP cells (but not non-SP cells) from REC-1 have been shown to repeatedly produce tumors in NOD/Shi-scid IL-2γnul mice containing both SP and non-SP cells in a serial transplantation assay [9]. PTC596 effectively decreased SP cell numbers by 97.7% at 150 nM, 99.8% at 300 nM, and > 99.9% at 600 nM compared with untreated controls (Figure 3). The reduction rates were much higher than that of ibrutinib (15.2% decrease even at 5 μM) (*P* < 0.0001), which currently is the most effective single agent against MCL. In comparison, the IC50 value for PTC596 was 138 nM in SP cells.

### PTC596 synergizes with the BTK inhibitor ibrutinib to induce mitochondrial apoptosis in MCL

**Figure 4:**
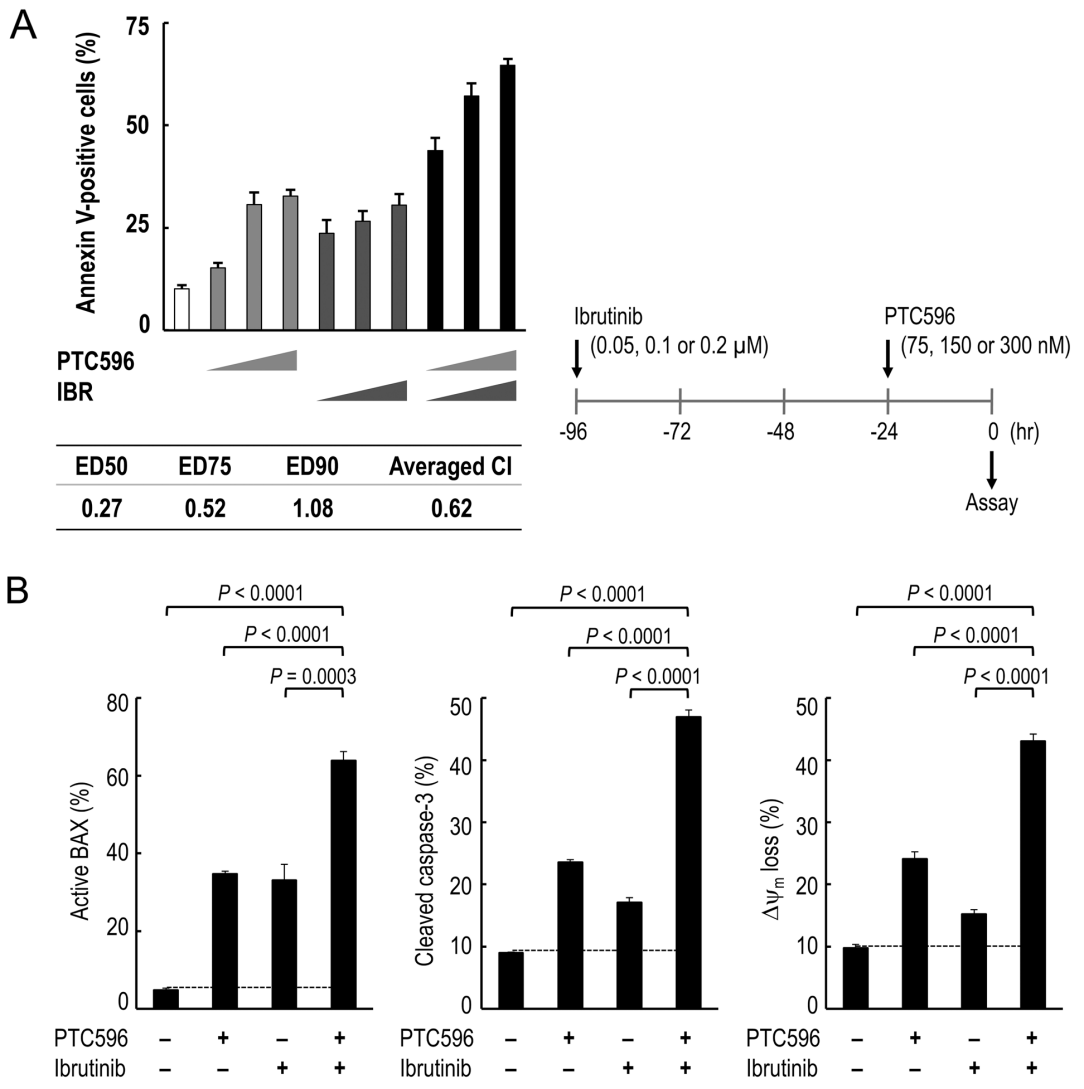
PTC596 and ibrutinib cooperatively induce mitochondrial apoptosis in mantle cell lymphoma cells **(A)** REC-1 cells were treated with PTC596 (75, 150, or 300 nM for 24 hours) and ibrutinib (IBR) (0.05, 0.1, or 0.2 μM for 96 hours) either as individual agents or in combination, after which the annexin V-positive fractions were determined. **(B)** REC-1 cells were treated for with PTC596 (150 nM, 24 hours) and ibrutinib (IBR) (0.1 μM, 96 hours) either as individual agents or in combination, after which BAX conformational changes, caspase-3 cleavage and Δψm loss were determined.

We investigated whether PTC596 could enhance the anti-lymphoma effects of chemotherapeutic agents that have been approved to treat patients with MCL. The combination index values for PTC596 with doxorubicin or bortezomib ranged from 1.0 to 1.2 (Table 3), indicating nearly additive or slightly antagonistic effects. The combination index values for PTC596 with cytarabine were 1.3 or higher, indicating antagonistic effects. We next investigated the potential therapeutic utility of combined molecular targeting of BMI-1 by PTC596 and BTK by ibrutinib. To investigate the nature of the interaction, we exposed B-cell receptor (BCR) signaling-activated and ibrutinib-sensitive REC-1 cells [24] to PTC596 (75, 150, or 300 nM for 24 hours) and/or low-dose ibrutinib (0.05, 0.1, or 0.2 μM for 96 hours), and evaluated phosphatidylserine externalization 24 hours after addition of PTC596. The interaction study showed synergistic effects on induction of phosphatidylserine externalization in REC-1 cells, with the low averaged combination index (CI) value of 0.62 (Figure 4A). The synergistic nature of the interaction was further confirmed in Z-138 cells that are less sensitive to ibrutinib [24] (Supplementary Figure 4).

**Table 3:** Combination index (CI) values for anti-proliferative and apoptotic effects

	Cell line	CI for anti-proliferative effect				CI for apoptotic effect			
		IC_50_	IC_75_	IC_90_	Averaged CI from IC_50_, IC_75_ and IC_90_	ED_50_	ED_75_	ED_90_	Averaged CI from ED_50_, ED_75_ and ED_90_
PTC596	Z-138	1.3	1.1	1.0	1.1	1.1	1.0	1.0	1.1
+ doxorubicin	JVM-2	0.7	0.9	1.4	1.0	1.1	1.0	1.0	1.0
	MAVER-1	1.1	1.0	1.0	1.0	1.0	1.0	1.0	1.0
PTC596	Z-138	1.2	1.1	1.1	1.1	1.2	1.2	1.1	1.2
+ bortezomib	JVM-2	0.8	0.9	1.2	1.0	1.1	1.2	1.2	1.2
	MAVER-1	1.3	1.2	1.1	1.2	1.1	1.2	1.3	1.2
PTC596	Z-138	1.7	1.4	1.3	1.5	1.5	1.5	1.6	1.5
+ cytarabine	JVM-2	1.0	1.2	1.8	1.3	1.9	2.4	3.0	2.4
	MAVER-1	2.2	1.3	0.9	1.5	1.6	1.9	2.6	2.3

Based on the data that PTC596 activates the intrinsic apoptosis pathway to kill MCL cells (Figure 2A, 2B), we investigated the combination effect of PTC596 and ibrutinib on intrinsic pathway activation. As shown in Figure 4B, ibrutinib augmented PTC596-induced BAX conformational changes, caspase-3 cleavage and Δψ_m_ loss in REC-1 cells. Similar synergistic interactions in intrinsic pathway activation were observed in Z-138 cells (Supplementary Figure 4B). Data suggest that PTC596 and ibrutinibcooperatively activate mitochondrial apoptosis signaling in MCL cells.

### PTC596 antagonizes ibrutinib-induced increase in MCL-1 expression and augments ibrutinib-induced apoptosis

**Figure 5:**
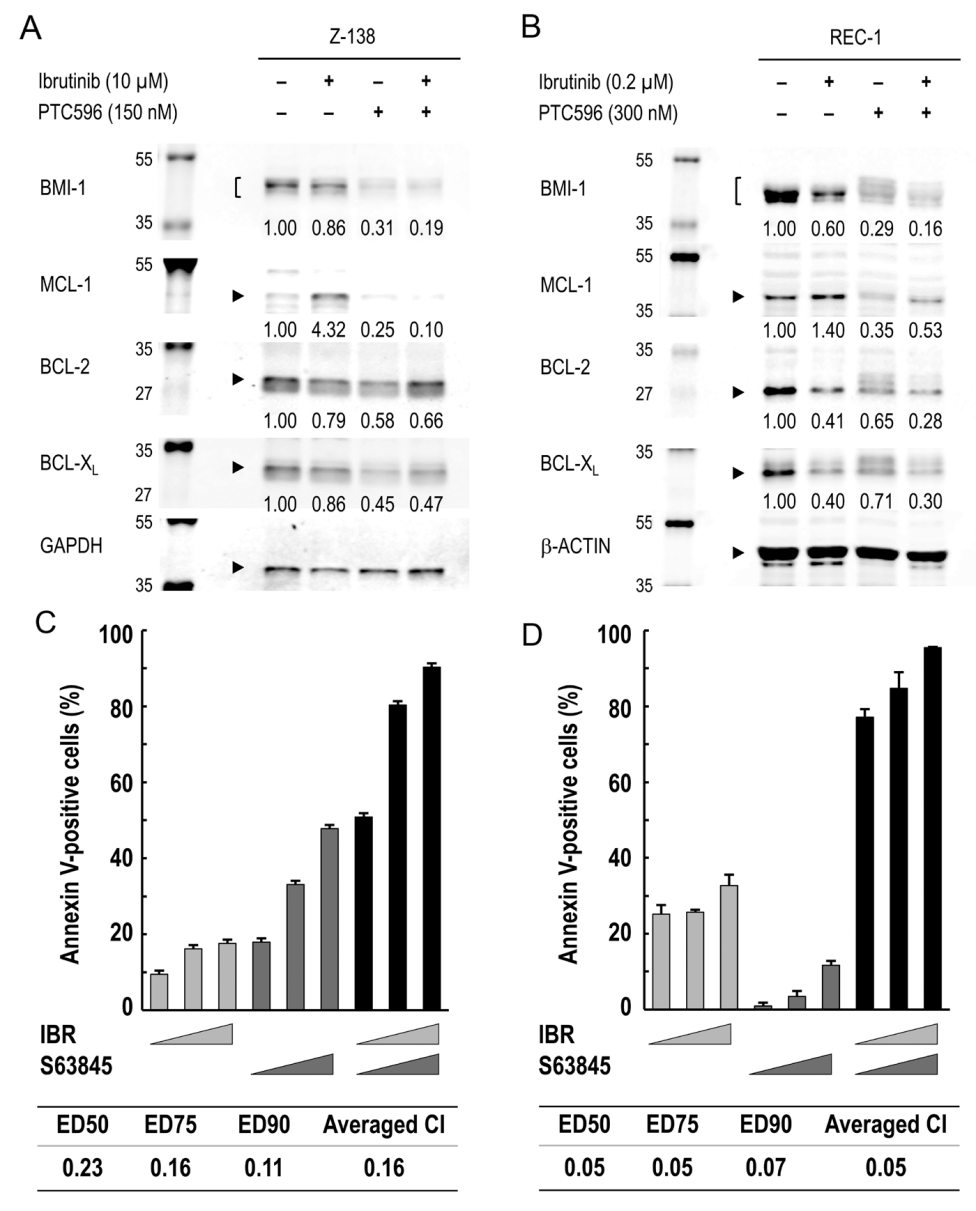
MCL-1 reduction by PTC596 counteracts ibrutinib-induced increase in MCL-1 expression and MCL-1 blockade augments ibrutinib-induced apoptosis **(A)** Z-138 cells were treated for 48 hours with 10 μM ibrutinib (IBR) and 150 nM PTC596 either as individual agents or in combination, after which BCL-2, BCL-X_L_ and MCL-1 protein levels were determined. **(B)** REC-1 cells were treated with IBR (0.2 μM, 96 hours) and PTC596 (300 nM, 24 hours) either as individual agents or in combination, after which BCL-2, BCL-X_L_ and MCL-1 protein levels were determined. PTC596 was added 72 hours after IBR exposure. **(C)** Z-138 cells were treated for 72 hours with IBR (2.5, 5 or 10 μM) and the selective MCL-1 inhibitor S63845 (100, 200 or 400 nM), either as individual agents or in combination, after which the annexin V-positive fractions were determined. **(D)** REC-1 cells were treated with IBR (0.05, 0.1 or 0.2 μM for 96 hours) and S63845 (50, 100 or 200 nM for 24 hours) either as individual agents or in combination, after which the annexin V-positive fractions were determined. The results are expressed as the mean ± SD.

To investigate the molecular mechanisms underlying the synergistic interaction of PTC596 with ibrutinib in inducing mitochondrial apoptosis, BCL-2, BCL-X_L_, and MCL-1 protein expression was determined in Z-138 and REC-1 cells. Unexpectedly, ibrutinib increased MCL-1 levels although it decreased BCL-2 and BCL-X_L_ levels (Figure 5A, 5B). PTC596 reduced MCL-1 expression levels (Figure 2C), which counteracted ibrutinib-induced increase in MCL-1 expression (Figure 5A, 5B). The hypothesis that MCL-1 is a synthetic lethal target in MCL cells under ibrutinib exposure was elucidated using S63845, a highly selective and potent MCL-1 inhibitor. As shown in Figure 5C, the ibrutinib/S63845 combination induced apoptosis in a highly synergistic fashion. Recent study suggests an association of PI3K–AKT pathway activation with the development of acquired ibrutinib resistance [6]. Since PI3K–AKT signaling upregulates MCL-1 expression [25, 26], we investigated if ibrutinib treatment leads to increased levels of phosphorylated AKT in Z-138 and REC-1 cells. As a result, levels of phosphorylated AKT did not change significantly after ibrutinib exposure (figure not shown), implying that PI3K–AKT pathway activation is not a primary mediator of MCL-1 upregulation in a short-term culture. The data suggested that PTC596 antagonizes ibrutinib-induced increase in MCL-1 expression and augments ibrutinib-induced apoptosis.

### PTC596 reduces BMI-1 and MCL-1 expression levels in SP cells

**Figure 6:**
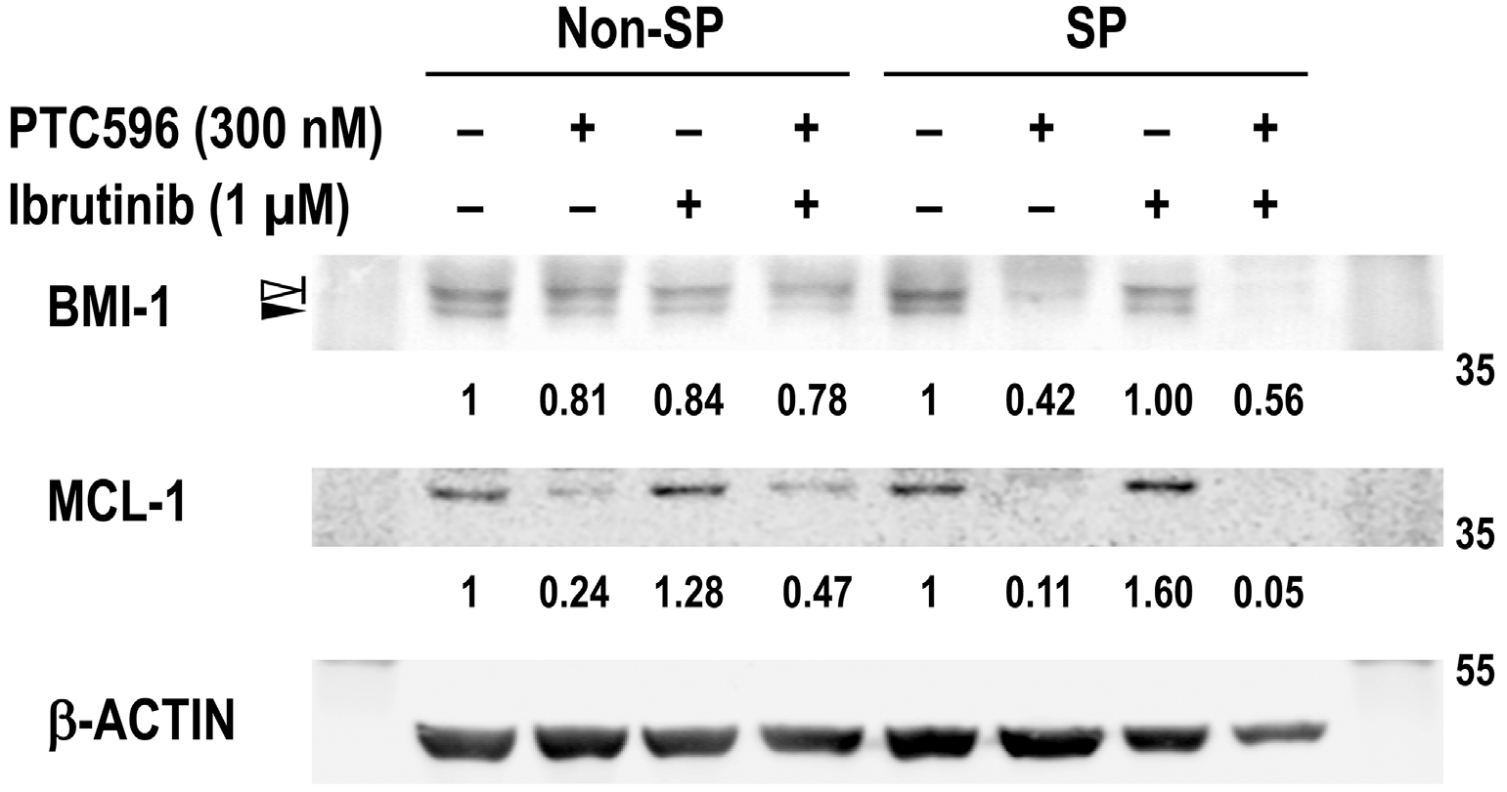
PTC596 depletes BMI-1 and MCL-1 expression in SP cells REC-1 cells were incubated for 24 hours with the indicated concentrations of ibrutinib and PTC596 either as individual agents or in combination. Viable cells were then sorted and collected into side population (SP) and non-SP cell populations, and BMI-1 and MCL-1 expression levels were determined. In BMI-1, slow migrating phosphorylated BMI-1 bands are indicated by the open arrowhead, and non-phosphorylated BMI-1 is indicated by the closed arrowhead. The intensities of immunoblot signals were quantified and normalized to those of β-ACTIN. Levels in untreated cells were set at 1.0.

BMI-1 has been actively involved in tumor-initiating SP cells in MCL [9] and we investigated if REC-1 SP cells are more susceptible to BMI-1 and MCL-1 reduction by PTC596 than non-SP cells. Basal BMI-1 levels were not significantly different between non-SP and SP cells in REC-1 cells (Figure 6). However, decreases in BMI-1 and MCL-1 levels after PTC596 treatment were more obvious in SP cells than non-SP cells. Although ibrutinib treatment led to slightly increased levels of MCL-1, the PTC596/ibrutinib combination almost completely diminished MCL-1 expression in SP cells. High susceptibility of SP cells to PTC596-mediated BMI-1 and MCL-1 reduction would provide the molecular rationale for the combination therapeutic strategy.

### Ibrutinib augments PTC596-induced apoptosis in primary cells from MCL and CLL patients

**Figure 7:**
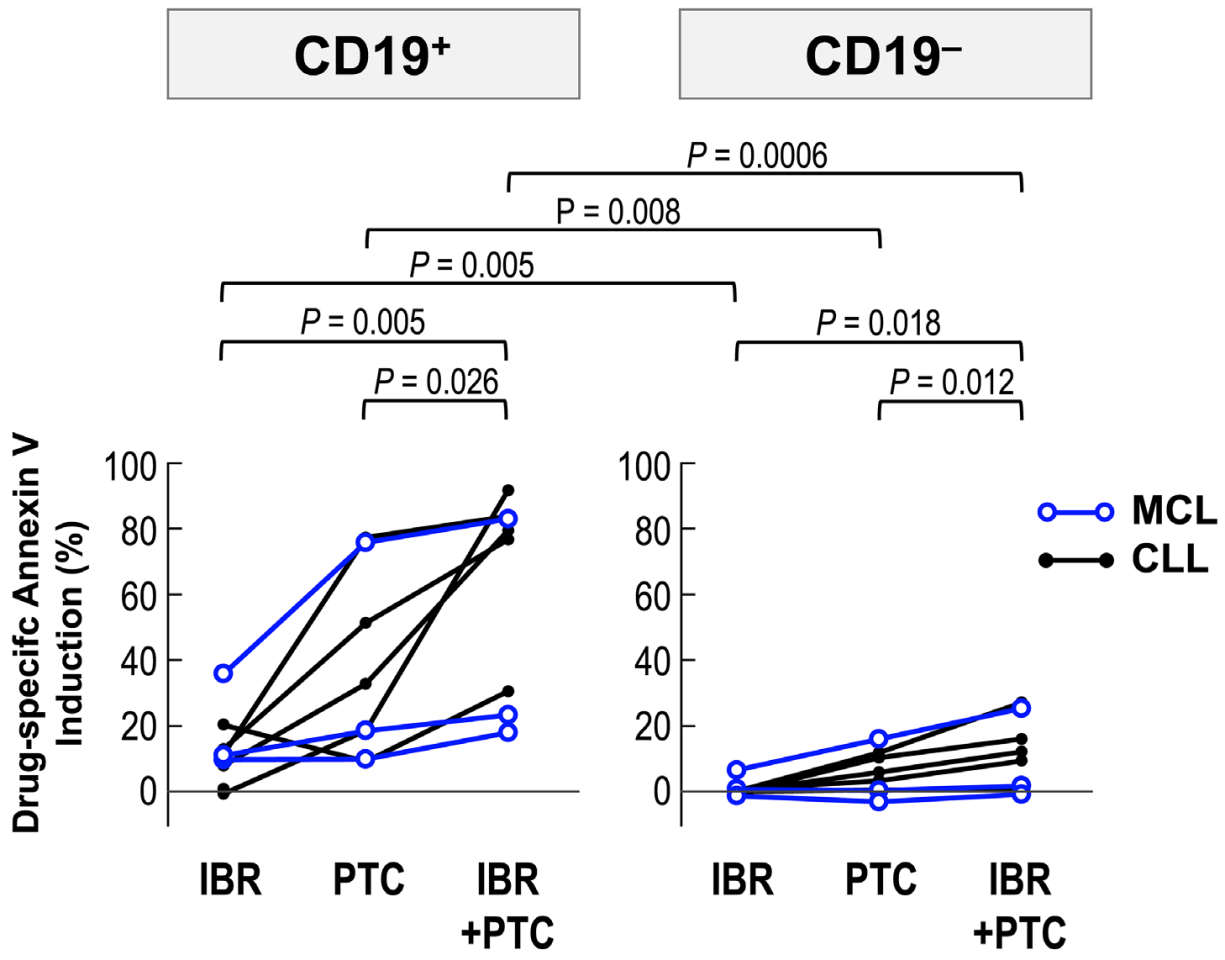
PTC596-induced apoptosis is enhanced by combination with ibrutinib in primary cells Primary MCL and CLL cells from patients were incubated for 24 hours with 2 μM ibrutinib and 2 μM PTC596, either as individual agents or in combination, and phosphatidylserine externalization was assessed by flow cytometry.

MCL and chronic lymphocytic leukemia (CLL) cells largely depend on B-cell receptor signaling for survival, and ibrutinib is indicated for the treatment of patients with these B-cell malignancies. We cultured primary samples with 2 μM ibrutinib and 2 μM PTC596 either as individual agents or in combination, and evaluated phosphatidylserine externalization after 24 hours. PTC596 induced phosphatidylserine externalization in CD19^+^ lymphoma/leukemia cells from all samples, although the effect varied widely among samples (Figure 7). Modest increases in annexin V-positive cells were found after exposure to ibrutinib. In both cases, CD19^+^ lymphoma/leukemia cells were much more susceptible than CD19^–^ normal cells. Ibrutinib augmented PTC596-induced phosphatidylserine externalization in all samples, which was prominent in CD19^+^ lymphoma/leukemia cell population. Data suggest that ibrutinib enhances PTC596-induced apoptosis in patient-derived samples.

## DISCUSSION

In this study, we investigated the therapeutic targeting of BMI-1 by the novel small molecule compund PTC596 in MCL. The proto-oncogene *BMI1* and its product, BMI-1, have been found to be upregulated in MCL, especially in aggressive variants and relapsed disease [9, 10]. Importantly, tumor-initiating MCL SP cells are reported to express significantly higher levels of BMI-1 than other cell fractions [9]. The clinical inhibitor of BMI1 expression, PTC596, showed potent anti-MCL activities at nanomolar concentrations. The concentration range of PTC596 used in this study was much lower than the plasma concentration observed in the Phase 1 clinical trial conducted in patients with advanced solid tumors, in which plasma concentrations reached 3.7 μM (Mean C_max_ of 1784 ng/mL) at the recommended Phase 2 dose [22]. All of the investigated MCL cell lines responded well to single-agent PTC596, irrespective of their reported BCR activation status or sensitivity to ibrutinib [24]. In addition, p53 status did not affect MCL susceptibility to PTC596. p53 abnormalities in MCL have been clinically associated with poor prognosis, and introduction of intensive chemoimmunotherapy has not been able to overcome its adverse prognostic value [27, 28]. Since intact p53 signaling is a critical mediator of conventional chemotherapy-induced cytotoxicity [29, 30], p53 independency in inducing apoptosis would be an advantage of the therapeutic targeting of BMI-1 by PTC596. Phase 2 studies should elucidate this hypothesis and if BMI-1 tumor expression could serve as a predictive biomarker for response.

The United States Food and Drug Administration approved ibrutinib for the treatment of patients with MCL who have received at least one prior therapy, based on a Phase 2 trial showing a 68% response rate with a median progression-free survival of 13.9 months [2]. Despite its dramatic initial responses, long-term remission remains elusive and acquisition of ibrutinib resistance has been universal [31]. Previously reported mechanisms of ibrutinib resistance in MCL are activating mutations of *BTK* and *CARD11* [32–34] and PI3K activation [6]. Although the major mode of action of ibrutinib has been thought to be a blockade of cellular responses to survival stimuli from the lymphoma microenvironment, our data raised the possibility that ibrutinib may not fully eradicate MCL tumor-initiating cells, leading to an increased risk of the proliferation of ibrutinib-resistant clones. As compared to ibrutinib, PTC596 effectively depleted SP cells at clinically relevant nanomolar concentrations. PTC596 and ibrutinib have the advantageous ability to kill MCL cells in a non-genotoxic manner. It has been suggested that the genotoxic chemotherapeutic agents may increase the risk of acquisition of new somatic mutations and development of chemoresistance or secondary primary malignancies in patients [35]. Since non-genotoxic small molecules PTC596 and ibrutinib synergistically induced apoptosis, this combination targeting lymphoma cells at a wide range of differentiation stages may represent a new treatment strategy for MCL.

It is clinically important that the cancer stem cell-targeting compound PTC596 decreased MCL-1 expression levels and antagonized ibrutinib-induced increase in MCL-1 expression. It has been reported that chemoresistant MCL cells commonly overexpress and are dependent on MCL-1 for survival [36, 37]. MCL-1 appears to be a critical effector of PTC596-induced apoptosis. In addition to the data presented in this study, we recently reported that PTC596 reduces MCL-1 expression in CD34^+^CD38^low/−^ stem/progenitor AML cells in association with apoptosis induction [14]. In contrast with PTC596 alone, ibrutinib treatment resulted in increased expression of MCL-1, which is in accordance with the recent data that ibrutinib induces kinome reprogramming, PI3K–AKT pathway activation and ibrutinib resistance in MCL [6]. Since short-term (up to 96 hours) ibrutinib exposure did not increase levels of phosphorylated AKT, we speculate that ibrutinib upregulates MCL-1 levels through multiple mechanisms. Although it remains unknown if long-term exposure of MCL cells to ibrutinib induces/selects MCL-1-overexpressing clones and leads to resistant disease, our data raise the possibility that BMI-1 inhibition not only targets tumor-initiating MCL cells but also counteracts proliferation of MCL-1-expressing ibrutinib-resistant clones.

In summary, our data indicate that the novel clinical candidate PTC596 induces mitochondrial apoptosis in BMI-1-expressing MCL cells in a p53-independent manner. MCL SP cells were susceptible to PTC596-induced apoptosis. Our findings strongly encourage the development of BMI-1-targeted therapy for MCL patients, especially for those with refractory or relapsed diseases that are frequently associated with BMI-1 overexpression. There are currently no drugs that specifically target cancer stem cell fractions, and a reduction in BMI-1 protein induced by PTC596 may offer a novel therapeutic strategy for MCL.

## MATERIALS AND METHODS

### Reagents

The selective small-molecule inhibitors of BMI-1, PTC-209 and PTC596 were provided by PTC Therapeutics, South Plainfield, NJ, USA. Ibrutinib was purchased from Santa Cruz Biotechnology (Santa Cruz, CA). S63845 was purchased from ChemieTek (Indianapolis, IN).

### Cells and cell culture

A total of eight MCL cell lines were used (Table 1). Z-138, JVM-2 and Granta-519 express wild-type p53, whereas MINO, JeKo-1, REC-1, MAVER-1 and NCEB-1 express mutant p53 [23, 38]. None of the eight cell lines have a *BMI1* gene amplification [39]. Cell lines were harvested in log-phase proliferation, seeded at a density of 1.5 × 10^5^ cell/mL and exposed to compounds. In the combination experiments of PTC596 with doxorubicin, bortezomib or cytarabine, the two agents were added simultaneously to Z-138, JVM-2, or MAVER-1 cells at a fixed concentration ratio and the cells were incubated for 72 hours. Cells were exposed to PTC596 at 1/4 ED50, 1/2 ED50, or ED50. The concentration ratio of doxorubicin to PTC596 was 1:20 in Z-138 and MAVER-1, and 1:40 in JVM-2 cells. The concentration ratio of bortezomib to PTC596 was 6:100 in Z-138 and JVM-2 cells, and 1:20 in MAVER-1 cells. The ratio was determined based on cell sensitivity to doxorubicin and bortezomib and their reported plasma concentration range in patients [40, 41]. The concentration ratio of cytarabine to PTC596 was 4:1 in Z-138, and 2:1 in JVM-2 and MAVER-1 cells, based on ED50 values for cytarabine. In experiments involving combination of PTC596 and ibrutinib in Z-138 cells, PTC596 (25, 50, or 100 nM) and ibrutinib (2.5, 5, or 10 μM) were added simultaneously and cultured for 72 hours. Ibrutinib-sensitive REC-1 [24] cells were exposed to ibrutinib for 72 hours before addition of PTC596 and then cultured for an extra 24 hours. Cell viability was evaluated by triplicate counts of trypan blue dye-excluding cells. Patient samples were analyzed under the protocol approved by the institutional review board at Saga University (2014-10-05). Heparinized peripheral blood samples were obtained from leukemic MCL and CLL patients after informed consent, according to institutional guidelines per the Declaration of Helsinki. Primary cells were cultured at 5 × 10^6^ /ml and treated with 5 μg/mL soluble goat F(ab’)2 anti–human IgM (Southern Biotechnology, Cambridge, UK).

### Flow cytometric analysis

For apoptosis analysis, flow cytometric determination of annexin V binding, conformational change in BAX, mitochondrial membrane potential loss (Δψ_m_), and caspase-3 cleavage were performed, as described previously [14].

### Western blot analysis

Western blot analysis was performed using the Odyssey imaging system (LI-COR Biosciences, Lincoln, NE) [23]. Antibodies against BMI-1, AKT, phospho-AKT (Ser473), PARP, GAPDH, and β-ACTIN were purchased from Cell Signaling Technology (Danvers, MA, USA), BCL-2 from Dako (Glostrup, Denmark), and BCL-X_L_ and MCL-1 from BD Biosciences.

### Quantitative real-time PCR

The mRNA expression levels were quantified using TaqMan gene expression assays (*BMI1*, Hs00995536_m1; *18S*, Hs99999901_s1) (Applied Biosystems, Foster City, CA, USA).

### SP analysis

To identify SP cells, cells were resuspended at a concentration 1 × 10^6^ cells/ml in Hank’s balanced salt solution supplemented with 2% fetal bovine serum, 2 mmol/l HEPES and 5 μg/ml Hoechst 33342 dye (Sigma-Aldrich, Darmstadt, Germany), and incubated for 60 min at 37 °C with intermittent shaking. SP cells were detected using a FACS Verse flow cytometer. Propidium iodide was used to discriminate live and dead cells. Verapamil (150 μM final concentration) was used to inhibit ATP-Binding Cassette transporters. SP and non-SP cells were respectively sorted by using a MoFlo Astrios EQ cell sorter (Beckman Coulter, Indianapolis, IN, USA).

### Statistical analyses

Statistical analyses were performed using a two-sided Student’s *t*-test, the non-parametric Mann–Whitney U-test, and the Pearson correlation coefficient as appropriate. Kaplan–Meier curves were used in combination with the log-rank test for survival analyses. A *P*-value of < 0.05 was considered statistically significant. Average values were expressed as means ± standard deviations (SD). The extent of apoptosis was quantified as percentage of annexin V-positive cells, and the extent of drug-specific apoptosis was assessed by the formula: % specific apoptosis = (test – control) x 100 / (100 – control) [42]. In the formula, the numerator is the actual amount of killing that occurred and the denominator is the potential amount of killing that could occur. The combination index (CI), a numerical description of combination effects, was calculated as described previously [43]. CI values indicate the following: <0.3, strong synergism; 0.3–0.7, synergism; 0.7–0.85, moderate synergism; 0.85–0.9, slight synergism; 0.9–1.1, nearly additive; 1.1–1.2, slight antagonism; 1.2–1.45, moderate antagonism; 1.45–3.3, antagonism; and >3.3, strong antagonism.

## SUPPLEMENTARY MATERIALS FIGURES


