# Supplementary material for: Targeting of BMI-1 expression by the novel small molecule PTC596 in mantle cell lymphoma
Source: Oncotarget. 2018 Jun 19;9(47):28547–60. doi: 10.18632/oncotarget.25558 (PMC6033370; doi:10.18632/oncotarget.25558)
Supplement: Supplementary file 1 [file oncotarget-09-28547-s001.pdf]

Targeting of BMI-1 expression by the novel small molecule PTC596 in mantle cell lymphoma

SUPPLEMENTARY MATERIALS

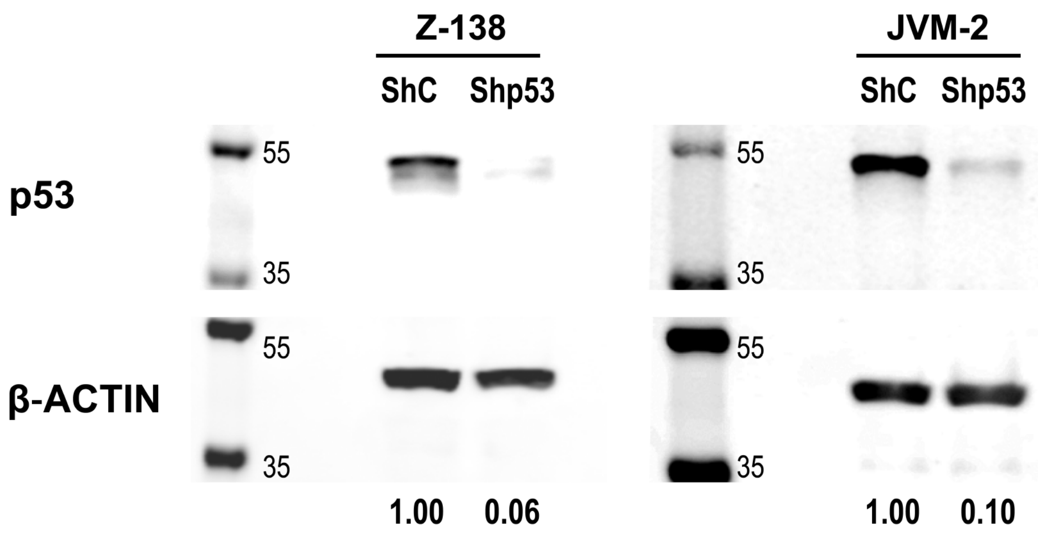

Supplementary Figure 1: *TP53*-shRNA reduced p53 basal levels by ~ 90% in MCL cell lines. p53 expression was determined in Z-138 and JVM-2, which were transduced with retroviruses encoding either scrambled shRNA (shC) or *TP53*-specific shRNA (shp53). Signal intensity was quantified and the relative intensity compared with β-ACTIN was calculated. Results are representative of three independent experiments.

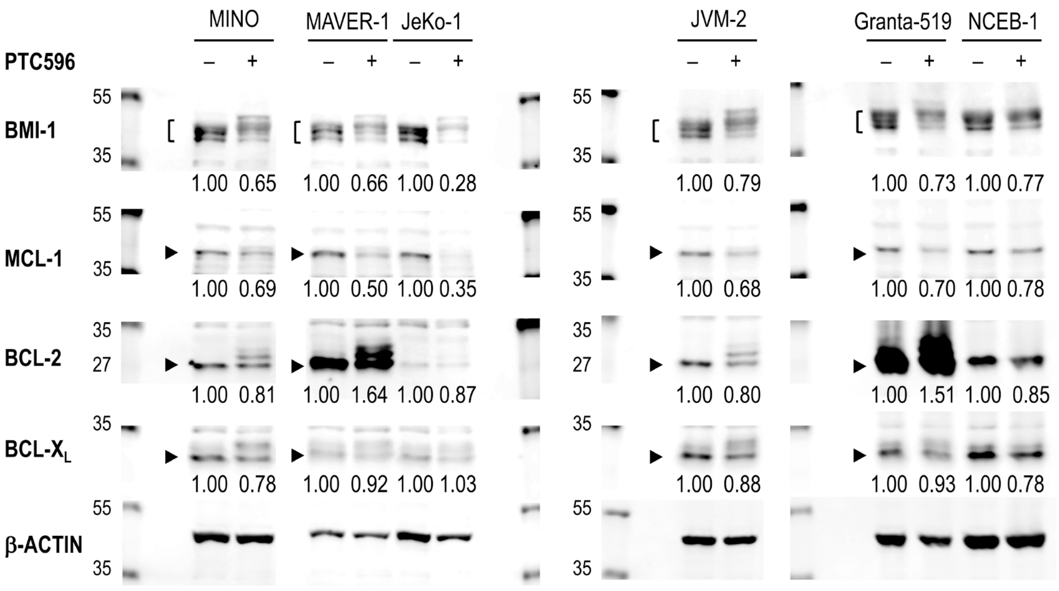

Supplementary Figure 2: MCL cells were treated for 24 hours with PTC596 (1 μM in relatively insensitive JVM-2 cells and 300 nM in other cells), after which BCL-2, BCL-X_L and MCL-1 protein levels were determined. The intensities of immunoblot signals were quantified and normalized to those of β-ACTIN. Levels in untreated cells were set at 1.0. Results are representative of three independent experiments.

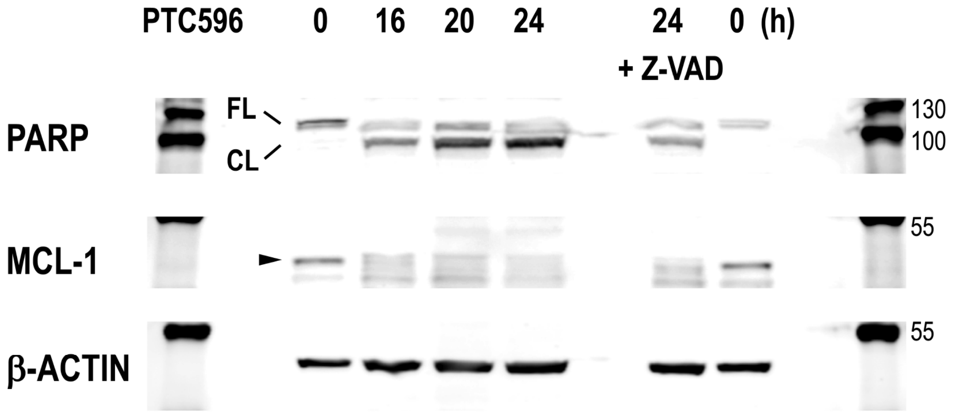

Supplementary Figure 3: Z-138 cells were treated with 300 nM PTC596 for the indicated times, in the absence or presence of 10 µM Z-VAD. Comparable results were obtained in two other independent experiments. FL, full length PARP. CL, cleaved PARP.

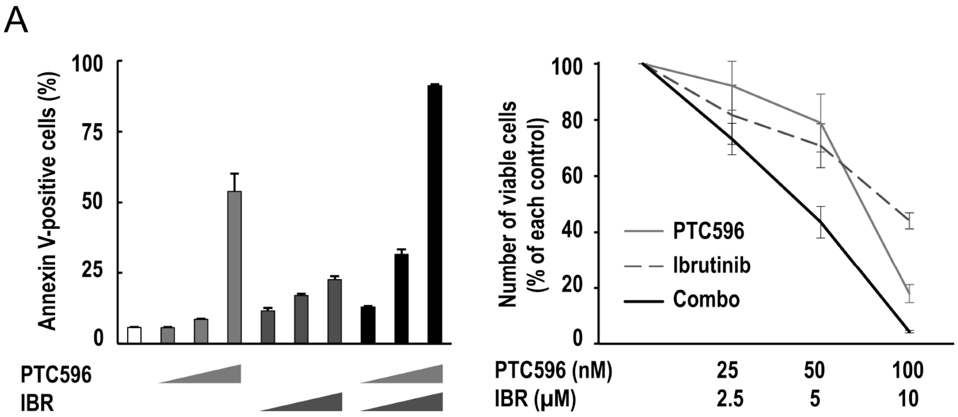

ED50	ED75	ED90	Averaged CI
0.66	0.66	0.71	0.68

IC50	IC75	IC90	Averaged CI
1.02	0.85	0.74	0.87

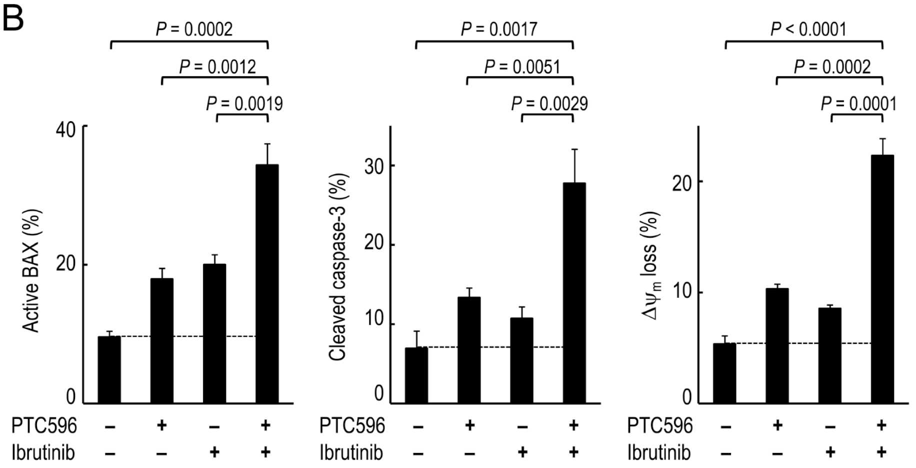

Supplementary Figure 4: PTC596 and ibrutinib cooperatively induce mitochondrial apoptosis in mantle cell lymphoma cells. **(A)** Z-138 cells were treated for 72 hours with PTC596 and ibrutinib (IBR) either as individual agents or in combination, after which the annexin V-positive fractions and the numbers of viable cells were determined. **(B)** BAX conformational changes, caspase-3 cleavage and Δψm loss were determined by flow cytometry in Z-138 cells after 24-h exposure to 100 nM PTC596 and 10 µM ibrutinib, either as individual agents or in combination. The results are expressed as the mean ± SD.
